# Commentary: Vasopressors in Early Burn Resuscitation: Physiological Concerns Revisited

**DOI:** 10.1111/aas.70270

**Published:** 2026-06-11

**Authors:** Folke Sjoberg, Jyrki Vuola, Andrew Lindford, Tina Palmieri, Moustafa Elmasry, Ahmed T. El‐Serafi, Ingrid Steinvall

**Affiliations:** ^1^ Department of Hand Surgery, Plastic Surgery, and Burns Linköping University Hospital Linköping Sweden; ^2^ Department of Biomedical and Clinical Sciences Linköping University (all in Linköping) Linköping Sweden; ^3^ Helsinki Burn Center Helsinki University Hospital and University of Helsinki Helsinki Finland; ^4^ Department of Burns, Shriners Children's Northern California and Department of Surgery University of California Davis, Sacramento California USA; ^5^ Department of Medical Laboratory Sciences University of Sharjah Sharjah UAE

The study by Knappskog et al. provides an important contribution to a persistent clinical controversy: the role of vasopressors during the early resuscitation phase following major burn injury [1]. Using a large multinational electronic health record database and rigorous propensity score matching, the authors demonstrate a consistent association between early vasopressor exposure and increased rates of acute kidney injury, sepsis, surgical intervention, and 30‐day mortality.

This large‐scale analysis is particularly valuable because it addresses a question frequently encountered in clinical practice but insufficiently supported by outcome data. Importantly, the observed associations are not unexpected; rather, they are highly consistent with established physiological principles of early burn shock and fluid resuscitation.

## 1 Physiological Context and Biological Plausibility

Early burn shock is characterized by profound fluid loss due to hydrostatic capillary effects, intravascular volume depletion, myocardial depression, and a compensatory increase in systemic vascular resistance. Effective resuscitation therefore focuses primarily on restoration of circulating volume while avoiding excessive crystalloid administration and its complications [2, 3].

Despite aggressive fluid therapy, patients remain hypovolemic during the initial phase until intravascular fluid volume begins to normalize, typically after 12–24 h [4, 5]. Neurohormonal activation during this period is pronounced, with elevated catecholamines, vasopressin, and angiotensin II contributing to increased vascular tone and redistribution of blood flow [6, 7].

Renal hypoperfusion during standard Parkland‐based resuscitation has been demonstrated by marked activation of the renin–angiotensin system even when urine output targets are achieved [8]. In this context, additional pharmacologic vasoconstriction may further compromise renal blood flow. The high incidence of early acute kidney injury observed in the vasopressor‐exposed cohort is therefore physiologically coherent and likely reflects the interaction between relative hypovolemia, endogenous vasoconstriction, and exogenous vasoactive therapy.

## 2 Microcirculation and Burn Wound Physiology

The skin is among the most α₁‐adrenergic receptor–dense vascular beds, and during early resuscitation it also becomes the principal interstitial reservoir for extravasated crystalloid. Edema‐related increases in interstitial pressure already impair tissue perfusion. Additional vasoconstriction may reduce blood flow within the zone of stasis, a mechanism long implicated in burn wound progression [9].

Experimental and clinical observations suggest that systemic vasoconstrictors can aggravate tissue ischemia and increase the need for surgical excision and grafting [10]. The higher operative rates reported in the vasopressor group are therefore consistent with known microcirculatory effects of catecholamines.

## 3 Interpretation of Outcome Associations

Patients receiving vasopressors are often more severely ill, and confounding by indication therefore remains a central limitation when interpreting these findings. Although the authors adjusted for major prognostic factors—including age, comorbidities, inhalation injury, and burn size—and the associations persisted after matching, residual confounding cannot be excluded. Important unmeasured factors such as the severity and trajectory of shock, adequacy and timing of fluid resuscitation, clinician thresholds for vasopressor initiation, vasopressor dose and duration, and evolving organ dysfunction may all have influenced both vasopressor exposure and outcomes.

The results should therefore be interpreted as clinically meaningful associations rather than evidence of a direct causal effect. Vasopressor use may represent a marker of early hemodynamic instability, delayed or insufficient volume resuscitation, or more severe circulatory dysfunction. Nevertheless, the consistency of the findings across clinically relevant outcomes strengthens their importance as hypothesis‐generating signals and supports careful physiological interpretation.

## 4 Clinical Implications and Knowledge Gaps

This study reinforces several established principles of burn resuscitation:

- Early burn shock is primarily a volume problem and, with the present guidelines, renders the patient hypovolemic for the first 18 h postburn.
- Vasopressors should not substitute for adequate intravascular resuscitation.
- Initiation during the hydrostatic capillary leak phase should be cautious and physiology‐guided.
- Strategies that minimize unnecessary early vasoconstriction may be beneficial.

At the same time, the analysis highlights critical gaps that also limit causal interpretation: the absence of information on vasopressor dose, timing, duration, shock severity, fluid balance, and clinician decision‐making. The dose–response relationship between vasopressor exposure and organ dysfunction remains unknown and will be difficult to address without prospective studies incorporating detailed hemodynamic, fluid balance, and microcirculatory data (American Burn Association Guidelines, 2023).

## 5 Conclusion

Rather than being surprising, the findings by Knappskog et al. [1] are consistent with long‐standing physiological concerns regarding early vasopressor use in burn resuscitation. However, because vasopressor‐treated patients are likely to represent a more severely ill subgroup, confounding by indication and residual confounding remain important limitations, and the observed associations should not be interpreted as proof of causality. By aligning large‐scale clinical outcomes with established burn pathophysiology, the study nevertheless provides important hypothesis‐generating evidence and underscores the need for cautious, volume‐first resuscitation strategies.

Future prospective investigations should focus on defining safe timing and dose thresholds for vasoactive therapy while accounting for shock severity, fluid balance, and evolving organ dysfunction. Until such data are available, this work supports a restrained and physiology‐guided approach to vasopressor use in the early management of patients with major burns.

## Author Contributions

All authors contributed equally to the conception, discussion, and formulation of the viewpoints expressed in this commentary. All authors reviewed and approved the final version of the manuscript.

## Funding

The authors have nothing to report.

## Conflicts of Interest

The authors declare no conflicts of interest.

## Data Availability

Data sharing is not applicable to this article as no datasets were generated or analysed during the current study.
